# Integrative Multi-Omics Analysis Unveils Candidate Genes and Functional Variants for Growth and Reproductive Traits in Duroc Pigs

**DOI:** 10.3390/ani15243627

**Published:** 2025-12-17

**Authors:** Zhuofan Yan, Xiyue Li, Wenbo Yang, Peng Zhou, Weiya Zhang, Xinyun Li, Liangliang Fu, Jingjin Li, Xiaoyong Du

**Affiliations:** 1 Key Laboratory of Agricultural Animal Genetics, Breeding and Reproduction, Ministry of Education, College of Animal Science and Technology, Huazhong Agricultural University, Wuhan 430070, China; zfyan@webmail.hzau.edu.cn (Z.Y.); wbyang_9527@webmail.hzau.edu.cn (W.Y.); zhou-peng@webmail.hzau.edu.cn (P.Z.); xyli@mail.hzau.edu.cn (X.L.); fuliangliang2017@mail.hzau.edu.cn (L.F.); jingjinli@webmail.hzau.edu.cn (J.L.); 2 College of Informatics, Huazhong Agricultural University, Wuhan 430070, China; 3 Key Laboratory of Intelligent Technology in Animal Husbandry, Ministry of Agriculture and Rural Affairs, Wuhan 430070, China; 4 Engineering Research Center of Smart Agricultural Technology, Ministry of Education, Wuhan 430070, China; 5 Hubei Province Research Center of Engineering Technology of Agricultural Big Data, Wuhan 430070, China; 6 College of Animal Science and Technology, Hebei Agricultural University, Baoding 071001, China; lixiyue198@163.com (X.L.); shyzwy@hebau.edu.cn (W.Z.); 7 Hubei Hongshan Laboratory, Frontiers Science Center for Animal Breeding and Sustainable Production, Wuhan 430070, China

**Keywords:** GWAS, pig, economic traits, TWAS, candidate gene

## Abstract

Pigs play a vital role in global food security as a major source of animal protein. Enhancing growth and reproductive traits is of great economic importance to the swine industry. To systematically identify genetic determinants underlying key economic traits, we performed an integrative multi-omics analysis in a population of 1624 Duroc pigs, focusing on backfat thickness (BF), loin muscle area (LMA), and total teat number (TTN). Our genome-wide association study (GWAS) identified twenty-one significant single nucleotide polymorphisms (SNPs)—fourteen for BF, three for LMA, and seven for TTN. Candidate genes located within 1 Mb of these SNPs, such as *ZC3HAV1L* and *FAM3A* for BF, *PTGR2* for LMA, and *VRTN* and *ABCD4* for TTN, were further investigated. Functional genomic annotations revealed that genetic variants near the significant SNPs were enriched in tissue-specific enhancer elements, implying regulatory potential. Transcriptome-wide association study (TWAS) further supported the candidate genes such as *ABCD4* and *YLPM1* for TTN and predicted several putative functional mutations that may affect transcription factor binding sites. This study demonstrates the power of integrative genomics to prioritize candidate genes and causal variants for animal complex traits, offering valuable resources for future precision breeding in pigs.

## Simple Summary

In pig breeding, improving carcass leanness, muscle yield, and reproductive performance is crucial for production efficiency. In this study, we investigated the genetic basis of three economically important traits—backfat thickness, loin muscle area, and total teat number—in 1624 Duroc pigs. Using genome-wide association and multi-omics analyses, we identified 21 significant genetic loci linked to these traits. Genes associated with fat deposition showed enrichment in adipose tissue regulatory regions, whereas genes influencing teat number were primarily active in intestinal tissues. For teat number, two key candidate genes, *ABCD4* and *YLPM1*, were highlighted, with functional variants predicted to alter transcription factor binding in hormone- and cholesterol-related pathways that are essential for mammary gland development. Our findings suggest potential molecular mechanisms underlying pig growth and reproduction and offer practical genetic markers to guide precision breeding for improved meat production and reproductive efficiency.

## 1. Introduction

Pigs are a foundation of global livestock production, and improving pork productivity remains essential for meeting increasing demand. Key economic traits—backfat thickness (BF), loin muscle area (LMA), and total teat number (TTN)—directly affect meat quality, carcass leanness, and sow productivity, thereby determining the efficiency and sustainability of swine production systems. These traits demonstrate moderate heritability (e.g., BF: ~0.37 [1,2]; LMA: 0.35–0.47 [3,4]) and are key breeding objectives in swine production, while TTN has been shown to play a critical role in regulating lactation capacity and piglet survival [5,6,7].

Genome-wide association studies (GWAS) have become a powerful tool for investigating such complex traits [8,9,10,11]. In pigs, GWAS has successfully identified numerous single nucleotide polymorphisms (SNPs) and candidate genes associated with growth and reproduction, such as *CACNA1E* and *ACBD6* for BF [12], and *VRTN* for TTN [13]. However, a major limitation persists: the majority of significant SNPs are located in non-coding regions, making it challenging to interpret their regulatory functions, identify causal mutations, and confirm effector genes [14,15,16]. The emergence of multi-omics resources offers a promising path forward. Functional genomic annotations, such as tissue-specific enhancer maps, can uncover the biological context in which non-coding variants function [17]. Simultaneously, transcriptome-wide association studies (TWAS) utilize expression quantitative trait loci (eQTL) to identify genes associated with traits, thereby establishing a direct link between genetic variation and gene function [18,19]. Platforms such as the FarmGTEx TWAS-Server have enabled such analyses across multiple major tissue types in pigs [20]. Although previous studies have provided valuable genetic insights into these traits [1,21,22], a systematic understanding of the key genes and their regulatory networks underlying BF, LMA, and TTN remains incomplete. This gap highlights the need to extend beyond GWAS through integrated multi-omics approaches.

**Figure 1:**
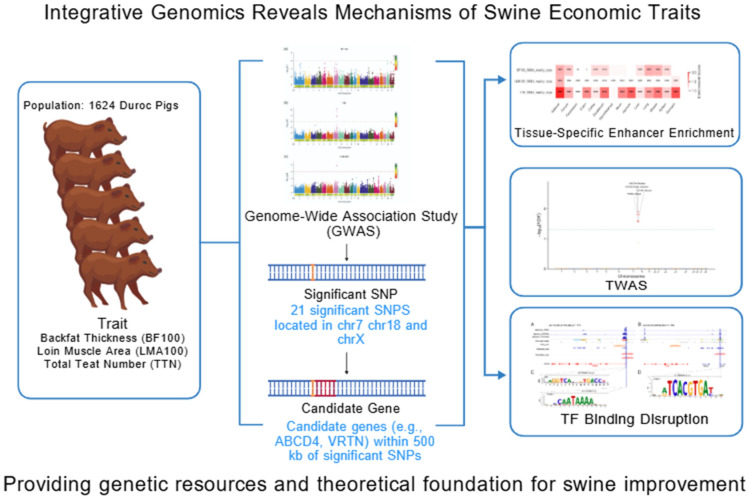
Workflow of the integrative multi-omics analysis. The schematic outlines the analytical pipeline. A population of 1624 Duroc pigs was phenotyped for BF100 (backfat thickness adjusted to 100 kg body weight), LMA100 (loin muscle area adjusted to 100 kg body weight) and TTN (total teat number). GWAS identified significant SNPs, from which candidate genes were selected. Convergent evidence from three complementary analyses—Tissue-Specific Enhancer Enrichment, TWAS, and TF Binding effect—was then integrated to prioritize high-confidence candidate genes and functional variants. This figure was created with BioGDP.com [23].

Therefore, to address this need, we conducted an integrative multi-omics analysis in a large population of Duroc pigs. We first performed GWAS for backfat thickness adjusted to 100 kg body weight (BF100), loin muscle area adjusted to 100 kg body weight (LMA100), and TTN, followed by tissue-specific enhancer enrichment analysis to explore the regulatory context of the associated variants. We then integrated TWAS to identify functional genes influencing these traits and further combined this with motif analysis to predict potential causal regulatory variants (Figure 1). This study aims to identify novel genetic variants, key candidate genes, and their regulatory mechanisms, thereby providing a scientific foundation for precision breeding in pigs.

## 2. Materials and Methods

### 2.1. Data Collection and Phenotyping

#### 2.1.1. Animal Population and Phenotypic Measurements

The study comprised a mixed cohort of 1624 Duroc pigs (964 males and 660 females) sourced from two commercial breeding companies in Guangxi and Hubei Provinces, China. All animals were raised under standardized conditions from 30 to 100 kg body weight, with uniform feed and management protocols to minimize environmental variability [24]. Backfat thickness (BF) and loin muscle area (LMA) at the 10th–11th rib were measured using an Aloka 500V SSD B ultrasound system (Corometrics Medical Systems, Wallingford, CT, USA) equipped with a linear transducer [25]. LMA values were quantified using proprietary image analysis software [26]. The measured BF and LMA were then adjusted to a standardized body weight of 100 kg (resulting in the traits BF100 and LMA100, respectively) using the correction formulas prescribed by the Chinese agricultural standard NY/T 2894-2016 [27].

#### 2.1.2. Genotyping and Data Processing

Genomic DNA was extracted from ear tissue using the phenol-chloroform method and genotyped using the CC1 Porcine SNP50 BeadChip [28]. Since the reference genome for the chip data in this study was *Sus scrofa* version 10.2, we utilized the UCSC Liftover tool (https://genome.ucsc.edu/cgi-bin/hgLiftOver (accessed on 1 September 2024)) to convert genomic coordinates from version 10.2 to 11.1 and checked allele consistency using bedtools [29]. The data were then converted to PLINK (v2.0) BED format for subsequent analyses.

### 2.2. Genome-Wide Association Study (GWAS)

#### 2.2.1. Genotype Data Quality Control and Population Structure Analysis

To ensure data quality, we performed SNP quality control using PLINK (v2.0), applying parameters --maf 0.01 and --mind 0.1 to exclude low-quality SNPs. After filtering, 1624 individuals and 43,800 SNPs were retained. The Genomic Relationship Matrix (GRM) was constructed using GCTA v1.93.2beta software [30], followed by principal component analysis (PCA) to explore population structure.

#### 2.2.2. Association Analysis

GWAS was performed using a mixed linear model (MLM) implemented in the R package MVP v1.4.5 [31]. To minimize potential confounding effects, several fixed covariates were included in the model, including sex, company, year, and month, which account for differences in biological characteristics and production management conditions. Population structure was controlled by incorporating the top five principal components (PCs) derived from PCA. The genome-wide significance threshold was set at *p* < 1.14 × 10^−6^ (Bonferroni correction: 0.05/43,800).

### 2.3. SNP Enrichment Analysis

To explore the tissues and biological functions potentially involved with genetic loci associated with the target traits, we conducted an enrichment analysis using two publicly available pig regulatory genomics resources. First, given the relatively low density of the SNP-chip data used in our GWAS and the need to account for linkage disequilibrium (LD) effects, we adopted a candidate-variant extension strategy using the whole-genome sequencing (WGS) variant dataset reported by Li et al. [32]. For each genome-wide significant SNP, a ±500 kb window upstream and downstream was defined, and all variants from the Li et al. [32] dataset falling within these windows were extracted to form an “extended candidate variant set.” Next, we evaluated whether these extended variants were enriched for regulatory annotations from Pan et al. [17], which mapped active enhancers across 14 major pig tissues. Enrichment analysis was performed using the Genomic Association Tester (GAT; https://gat.readthedocs.io/ (accessed on 20 October 2025)) [33].

### 2.4. Candidate Gene Identification and Functional Annotation

Significant SNPs (*p* < 1.14 × 10^−6^) were annotated using the Ensembl database (Release 110). Genes located within 500 kb upstream or downstream of significant SNPs were prioritized, while additional candidate genes related to pig growth and reproduction were supplemented through literature mining. Gene Ontology Biological Processes (GO BP) and KEGG pathway enrichment analyses for BF100, LMA100, and total teat number (TTN) were conducted using Metascape (http://metascape.org (accessed on 20 October 2025)) with parameters: Min Overlap = 3, *p*-value cutoff = 0.01, and Min Enrichment = 1.5.

### 2.5. TWAS Analysis

To provide transcriptomic references for candidate genes and identify the primary tissues in which they act, cross-tissue transcriptome-wide association analysis (TWAS) was performed using the FarmGTEX platform (https://www.farmgtex.org/ (accessed on 10 September 2025)) [34,35]. GWAS summary statistics for BF100, LMA100, and TTN were used as input for imputation via the MetaXcan algorithm implemented on the FarmGTEX TWAS-Server. Expression reference models from 34 pig tissues (including adipose, liver, muscle, ovary, testis, brain, heart, and others) were integrated for cross-tissue analysis. Bonferroni-corrected significance thresholds were calculated as −log_10_(0.05/N), where N is the total number of tested genes.

### 2.6. Identification of Candidate Regulatory Variants

To pinpoint putative regulatory variants that may drive GWAS associations, we implemented a stepwise screening strategy. For each significant SNP, we first determined whether any TWAS significant gene resided within a ±500 kb flanking region. For each SNP meeting this criterion, we extracted all variants within a ±500 kb window centered on the GWAS SNP from the WGS variant dataset reported by Li et al. [32]. From this set of variants, we retained only those that directly overlapped with the promoter or enhancer elements defined in the porcine functional genomic atlas of Pan et al. [17]. These overlapping variants were subjected to subsequent transcription factor (TF) motif analysis.

### 2.7. TF Motif Analysis

To evaluate the potential regulatory impact of the variants identified in Section 2.6, we performed TF motif analysis using the R package motifbreakR [36]. The variants obtained from the overlap-based filtering in Section 2.6 were used as input. For each variant, motifbreakR predicted its potential effect on TF binding. A *p*-value threshold of 1 × 10^−4^ was applied to identify statistically significant effects, and only those classified as having a “strong” impact were retained for further interpretation.

## 3. Results

### 3.1. Phenotypic and Genotypic Data Summary

Summary statistics for backfat thickness adjusted to 100 kg body weight (BF100), loin muscle area adjusted to 100 kg body weight (LMA100), and total teat number (TTN) are presented in Table 1. A total of 1546 individuals were phenotyped for BF100 (mean = 9.74 cm) and LMA100 (mean = 49.49 cm^2^), and 1235 individuals were phenotyped for TTN (mean = 13.64). Moderate-to-high coefficients of variation (CV) were observed for BF100 (19.11%) and LMA100 (21.43%), reflecting substantial phenotypic variability conducive to genetic analyses. In contrast, TTN exhibited limited variation (CV = 7.74%), suggesting relatively stable expression of this reproductive trait within the population.

Genotype data were obtained from 1624 individuals (1546 for BF100 and LMA100, 1235 for TTN), with a total of 43,800 SNPs after quality control. SNP density plots for each trait are shown in Supplementary Figure S1. Principal component analysis (PCA) revealed clear clustering patterns among individuals (Supplementary Figure S2). For the BF100 and LMA100 datasets (n = 1546), samples separated into three distinct genetic clusters. In contrast, the TTN dataset (n = 1235) formed two primary clusters. These clusters corresponded closely to the two commercial origins of the pigs. This population structure was effectively controlled for in all subsequent association models.

### 3.2. Genome-Wide Association Analysis (GWAS) of BF100, LMA100, and TTN

**Figure 2:**
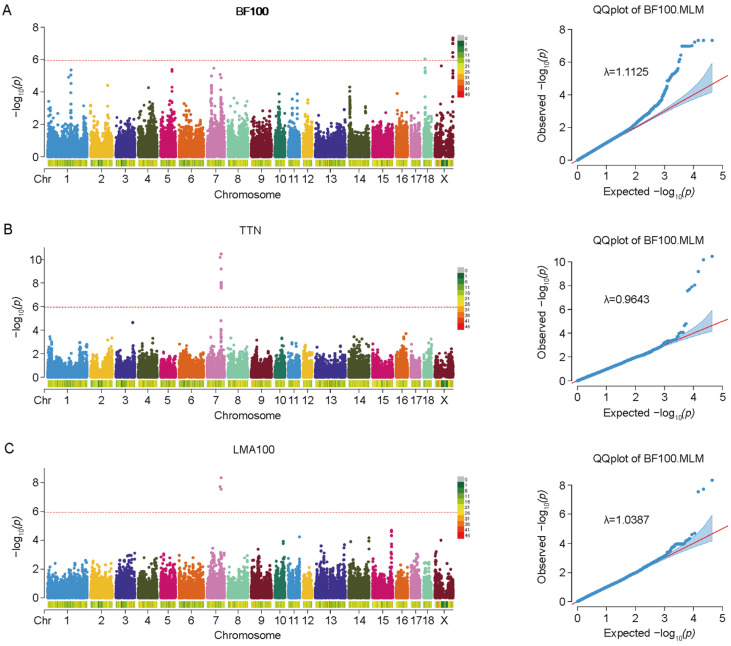
Manhattan plots and Q-Q plots of SNP dominance effects for three traits: (**A**) BF100 (backfat thickness adjusted to 100 kg body weight), (**B**) TTN (total teat number), and (**C**) LMA100 (loin muscle area adjusted to 100 kg body weight). The red dotted line indicates the potential significance threshold.

GWAS using a mixed linear model identified 21 significant SNPs across the three traits after Bonferroni correction (Figure 2). The genomic inflation factors (λ) were 1.1125 for BF100, 1.0387 for LMA100, and 0.9643 for TTN, indicating adequate control of population structure.

Across the three traits, a total of 21 genome-wide significant SNPs were identified—14 for BF100, 3 for LMA100, and 7 for TTN. For BF100, the 14 significant SNPs were predominantly located on the X chromosome, with the strongest signal observed at rs327767193 within the *FAM3A* gene. Three SNPs on chromosome 7 (Chr7:91308348:G:T, rs330032123, and rs711580469) were found to be associated with both LMA100 and TTN.

The strongest of these overlapping signals, rs711580469, mapped to the *VRTN* gene, a key regulator of vertebral development conserved across species [37,38]. Additionally, seven TTN-associated SNPs clustered on chromosome 7, including the lead SNP rs330032123 within *ABCD4*, flanked by *VSX2* and *VRTN*.

Candidate genes located within ±0.5 Mb of these significant SNPs are summarized in Table 2.

### 3.3. Enrichment Analysis of Trait-Associated SNPs

To investigate the tissue-specific regulatory architecture underlying the target traits, we performed enrichment analyses using the extended candidate variant sets derived from our GWAS results. Each set was constructed by extending genome-wide significant SNPs by ±500 kb and incorporating all variants from the whole-genome sequencing (WGS) variant dataset reported by Li et al. [32], thereby accounting for LD effects and capturing nearby regulatory variants. The functional relevance of these extended variants was then assessed by testing their overlap with enhancer annotations across 14 major pig tissues, as defined in the pig functional genome atlas reported by Pan et al. [17]. Enrichment was evaluated using the Genomic Association Tester (GAT; https://gat.readthedocs.io/ (accessed on 22 April 2025)) [33].

**Figure 3:**
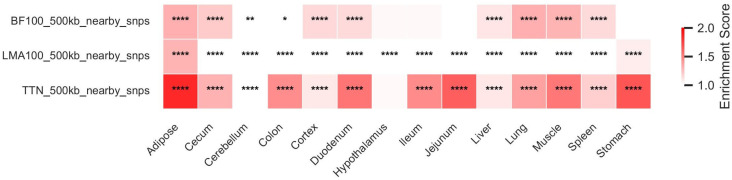
Enrichment of genetic variants for pig economic traits in tissue-specific enhancers. The heatmap displays the enrichment scores of candidate regions (within ±500 kb of significant SNPs) associated with backfat thickness (BF100), loin muscle area (LMA100), and total teat number (TTN) across enhancers from 17 distinct tissues. Enrichment analysis was performed using the Genomic Association Tester (GAT). The significance markers in the cells are categorized based on *p*-values: **** (*p*-values ≤ 0.0001), ** (*p*-values ≤ 0.01), * (*p*-values ≤ 0.05), with no marker indicating no significant enrichment. The full set of enrichment statistics is available in Supplementary Table S1.

Distinct tissue enrichment patterns were observed for the three economic traits (Figure 3). Adipose tissue showed significant enrichment for all three traits, suggesting that regulatory variants influencing BF100, LMA100, and TTN are all partially mediated through adipose-related regulatory mechanisms.

The complete statistical details, including *p*-values and false discovery rates (FDR), are provided in Supplementary Table S1.

### 3.4. TWAS Reveals Functional Genes and Underlying Regulatory Variants for TTN

**Figure 4:**
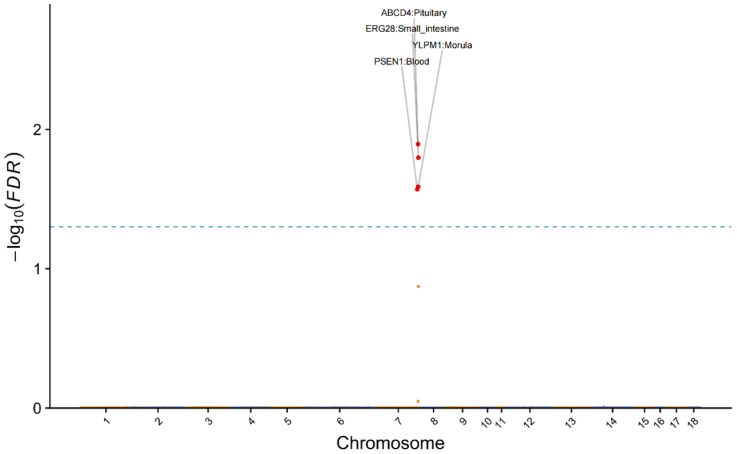
Manhattan plot of transcriptome-wide association study (TWAS) for total teat number (TTN). The *y*-axis indicates the association significance (−log_10_(FDR)). The dashed line indicates the multiple testing-corrected significance threshold (FDR < 0.05).

**Figure 5:**
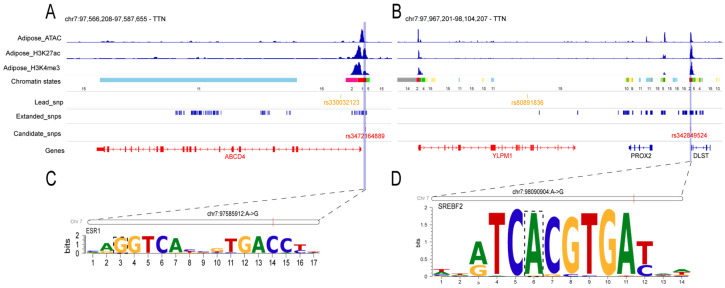
Predicted regulatory mechanisms and candidate functional mutations for TTN-associated genes. (**A**,**B**) Genomic views of two loci on chromosome 7 show adipose epigenomic profiles and GWAS SNPs, identifying *ABCD4* and *YLPM1* as candidate genes. The top four tracks display epigenomic profiles and chromatin state annotations (ChromHMM 15-state model) from Pan et al. [17], where numeric states (1–15) correspond to active promoters (TssA, TssAHet), transcriptional regions (TxFlnk, TxFlnkWk, TxFInkHet), enhancers (EnhA, EnhAMe, EnhAWk, EnhAHet, EnhPois), open chromatin (ATAC_Is), bivalent or repressed promoters (TssBiv, Repr, ReprWk), and quiescent regions (Qui). (**C**,**D**) Transcription factor (TF) motif analysis predicts candidate SNPs alter binding of key transcription factors: ESR1 at the *ABCD4* promoter (**C**) and SREBF2 downstream of *YLPM1* (**D**).

To identify genes whose expression is associated with TTN, we performed a transcriptome-wide association study (TWAS) using the FarmGTEx TWAS-Server [20]. The analysis integrated expression prediction models across 34 tissues, including metabolic, reproductive, neural, and systemic tissue types (see Methods). The cross-tissue TWAS Manhattan plot for TTN is shown in Figure 4, highlighting significant genes and their primary associated tissues. The analysis identified four genes significantly associated with the TTN trait: *ABCD4*, *ERG28*, *PSEN1*, and *YLPM1*. The association for *ABCD4* was detected in pituitary tissue, a central endocrine gland that regulates mammary development via hormones such as prolactin [39], suggesting that genetic variation influencing *ABCD4* expression may affect teat development via this endocrine pathway. Similarly, the association of *YLPM1* with morula—an early embryonic stage—suggests that its genetic regulation may be established during early development and exert lasting effects on processes relevant to later mammary morphogenesis. These TWAS signals were used to prioritize candidate regulatory variants for further functional investigation (Figure 5).

For the *ABCD4* gene, which is involved in vitamin B12 metabolism [40]—a pathway with implications for lipid metabolism [41]—we identified a candidate regulatory variant, rs3472164889, within a promoter region marked by active epigenomic modifications in the jejunum (Figure 5A). Motif analysis predicted that this variant may affect the binding of transcription factor ESR1 (Figure 5C). Experimental studies have shown that reduced ESR1 expression in the mammary gland correlates with alterations in glandular morphology [42].

For the *YLPM1* gene, our analysis highlighted a candidate functional mutation, rs342849524, located 48.62 kb downstream of the gene within a promoter region (Figure 5B). *YLPM1* plays a key role in cellular differentiation by repressing telomerase activity through binding to the TERT promoter [43]. Motif analysis predicted that this mutation may affect transcription factor SREBF2, which has been confirmed as a sterol-regulatory element-binding transcription factor with significant effects on lipid synthesis [44,45] (Figure 5D). 

### 3.5. Functional Enrichment Analysis of Candidate Target Genes

**Figure 6:**
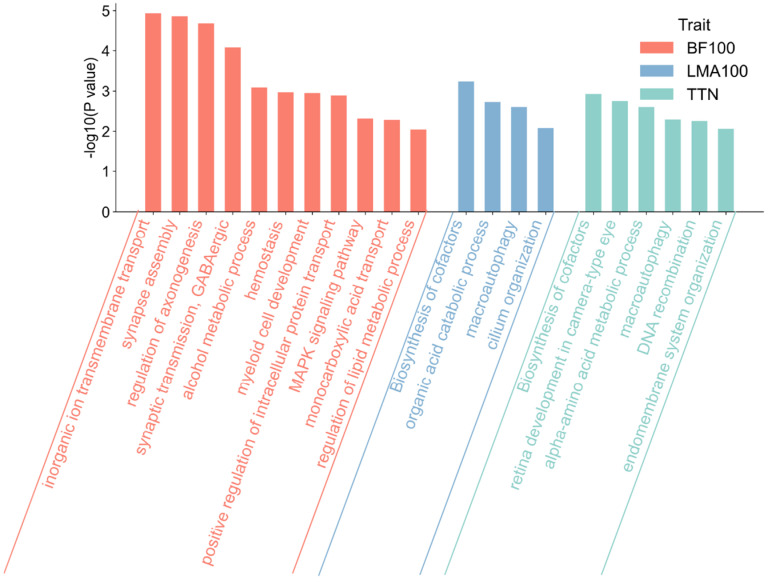
GO and KEGG enrichment analysis of candidate genes associated with BF100, LMA100, and TTN traits.

To characterize the biological pathways underlying the identified candidate genes, Gene Ontology (GO) Biological Process and KEGG pathway enrichment analyses were performed for BF100-, LMA100-, and TTN-associated genes using Metascape (Figure 6). Genes associated with BF100 were significantly enriched in processes related to neurodevelopment, such as synapse assembly and axonogenesis, and in the MAPK signaling pathway, which has known roles in adipogenesis regulation. Genes associated with LMA100 were enriched in pathways involved in cellular metabolism and structural organization, including organic acid catabolic process, macroautophagy, and cilium organization. In contrast, TTN-associated genes were primarily enriched in cofactor biosynthesis and amino acid metabolic processes.

Collectively, these results reveal distinct biological processes and molecular pathways contributing to the regulation of growth and reproductive traits in pigs.

## 4. Discussion

Duroc pigs are a key genetic resource in global swine breeding, with traits such as backfat thickness (BF), loin muscle area (LMA), and total teat number (TTN) directly affecting production efficiency and economic returns. However, the functional regulatory mechanisms underlying these traits remain incompletely understood. In this study, we investigated their genetic basis by integrating genome-wide association studies (GWAS), SNP enrichment analysis, transcriptome-wide association studies (TWAS), and transcription factor (TF) motif analysis. Through this multi-level genomic approach, we aimed to identify key loci and functional regulatory elements underlying phenotypic variation in these traits, thereby providing a deeper understanding of their underlying genetic mechanisms.

Applying this approach, we investigated the functional context of significant associations. For backfat thickness adjusted to 100 kg body weight (BF100), GWAS signals near adipogenesis-related genes (*ZC3HAV1L*, *FAM3A*) [46,47] were further supported by their enrichment in adipose-specific enhancers. This suggests that the genetic influence on BF100 is mediated, at least in part, by variants that modulate regulatory activity specifically in adipose tissue. This moves beyond mere statistical association to implicate specific tissue-level regulatory mechanisms. Notably, genetic variants associated with BF100, loin muscle area adjusted to 100 kg body weight (LMA100), and TTN all showed enrichment in adipose tissue enhancers, suggesting adipose-derived signals may serve as a common regulatory node influencing these distinct traits.

Our TWAS and subsequent regulatory analyses nominated *ABCD4* and *YLPM1* as prioritized candidate genes for TTN. For *ABCD4*, the promoter variant rs3472164889 is predicted to affect the binding of ESR1, a key transcriptional regulator in mammary biology. Reduced ESR1 expression has been experimentally linked to altered mammary gland morphology [42]. Independently, functional studies indicate that *ABCD4* can modulate mammary epithelial cell proliferation and apoptosis, and upregulate the prolactin receptor (*PRLR*) [48]. For *YLPM1*, the downstream variant rs342849524 is predicted to influence the binding of SREBF2, a master transcriptional regulator of cholesterol and fatty acid synthesis [44,45]. Given that cholesterol is the essential precursor for all steroid hormones, genetic variation at this locus could modulate the metabolic substrate pool available for hormone biosynthesis, thereby potentially influencing mammary gland development. Furthermore, *YLPM1* itself has been reported to regulate cellular differentiation, including through the repression of telomerase activity [43]. This suggests that *YLPM1* may also affect mammary development by modulating the proliferation or differentiation state of mammary epithelial cells, complementing its potential role in lipid-mediated hormonal regulation. Notably, the TWAS signal for *YLPM1* originated from morula, indicating that its genetic regulation is established early and may exert lasting influence on cell fate decisions pertinent to mammary morphogenesis.

Several limitations should be considered when interpreting our findings. First, the moderate density of the SNP chip limited fine-mapping resolution, and the absence of genotype imputation may have reduced the detection of trait-associated signals. Second, the lack of a population-matched, multi-tissue expression quantitative trait loci (eQTL) reference precluded formal colocalization analysis (e.g., using COLOC), which would be necessary to strengthen causal inference for TWAS-identified genes such as *ABCD4* and *YLPM1*. Future studies should prioritize: (1) high-depth whole-genome sequencing in expanded Duroc populations to improve mapping precision; (2) development of breed-specific multi-tissue eQTL resources; and (3) experimental validation, both in vitro and in vivo, of the functional roles of candidate genes identified in this study.

## 5. Conclusions

This study employed an integrative multi-omics approach to dissect the genetic architecture of key economic traits in Duroc pigs. A total of 21 significant SNPs and several candidate genes were identified, such as *FAM3A* and *ZC3HAV1L* for backfat thickness. Furthermore, by integrating transcriptome-wide association and motif analyses, *ABCD4* and *YLPM1* were implicated as candidate genes of teat number. Collectively, these findings provide a set of supported candidate genes and variants, establishing a foundation for future functional studies and molecular breeding applications in pigs.

## Figures and Tables

**Table 1:** Descriptive statistics of phenotypic traits in the Duroc population.

Trait	Effective Record Count	Mean	Standard Deviation	Maximum	Minimum	Coefficient of Variation (%)
BF100 (cm)	1546	9.74	1.86	20.30	5.38	19.11
LMA100 (cm^2^)	1546	49.49	10.61	71.64	25.76	21.43
TTN	1235	13.64	1.06	16.00	10.00	7.74

**Table 2:** Summary of significant SNPs and candidate genes associated with BF100, LMA100, and TTN in GWAS.

Traits	SNP ID	Chrom	Position	*p*-Value	Candidate Genes
BF100	rs81236473	18	10555467	9.34 × 10^−7^	*ZC3HAV1L*
	rs81333323	X	123188729	3.72 × 10^−7^	*CNGA2\GABRE*
	rs332246267	X	123225920	1.06 × 10^−7^	*CNGA2\GABRE*
	rs329206246	X	123251807	1.06 × 10^−7^	*CNGA2\GABRE*
	rs336224043	X	123302262	1.06 × 10^−7^	*CNGA2\GABRE*
	rs334203110	X	123655172	1.06 × 10^−7^	*GABRA3*
	rs320267534	X	123827689	1.06 × 10^−7^	*BGN/SLC6A8*
	rs80918182	X	123849200	1.06 × 10^−7^	*BGN/SLC6A8*
	rs330196368	X	123955387	9.97 × 10^−8^	*BGN/SLC6A8*
	rs326373823	X	124344831	6.02 × 10^−8^	*BGN/SLC6A8*
	rs81245332	X	124482822	6.78 × 10^−7^	*BGN/SLC6A8*
	rs330863063	X	124702511	4.72 × 10^−8^	*SLC6A8/FAM3A*
	rs327767193	X	125021305	4.72 × 10^−8^	*FAM3A*
	rs344761734	X	125135139	4.72 × 10^−8^	*FAM3A*
LMA100/TTN	/	7	91308348	1.93 × 10^−8^	*PIGH*
	rs330032123	7	97584287	2.91 × 10^−8^	*PTGR2/FAM161B/LIN52/ABCD4/VRTN/SYNDIG1L/LTBP2/AREL1/YLPM1/PROX2*
	rs711580469	7	97622770	4.54 × 10^−9^	*PTGR2/FAM161B/LIN52/ABCD4/VRTN/SYNDIG1L/LTBP2/AREL1/YLPM1/PROX2/DLST*
TTN	rs81396056	7	97732109	1.95 × 10^−8^	*ABCD4/NPC2/VRTN/SYNDIG1L/LTBP2/AREL1/YLPM1/PROX2/DLST*
	rs80891836	7	98022168	1.25 × 10^−8^	*ABCD4/NPC2/VRTN/SYNDIG1L/LTBP2/AREL1/YLPM1/PROX2/DLST*
	rs80836267	7	98089286	8.95 × 10^−9^	*VRTN/SYNDIG1L/LTBP2/AREL1/YLPM1/PROX2/DLST*
	rs336641062	7	98116120	2.68 × 10^−8^	*VRTN/RPS6KL1/SYNDIG1L/LTBP2/AREL1/YLPM1/PROX2/DLST*

## Data Availability

The raw data supporting the conclusions of this article will be made available by the authors on request.

## Supplementary Materials

The following supporting information can be downloaded at: https://www.mdpi.com/article/10.3390/ani15243627/s1, Figure S1: SNP Density Plots for BF100, TTN, and LMA100 Traits; Figure S2: Principal Component Analysis (PCA) of Genomic Variation in Duroc Pigs. Table S1: Complete statistical results of the tissue-specific enhancer enrichment analysis for BF100, LMA100, and TTN.
