# Supplementary material for: Integrative Multi-Omics Analysis Unveils Candidate Genes and Functional Variants for Growth and Reproductive Traits in Duroc Pigs
Source: Animals (Basel). 2025 Dec 17;15(24):3627. doi: 10.3390/ani15243627 (PMC12730100; doi:10.3390/ani15243627)
Supplement: Supplementary file 1 [file animals-15-03627-s001.zip › supplementary figures.pdf]

Supplementary Figures

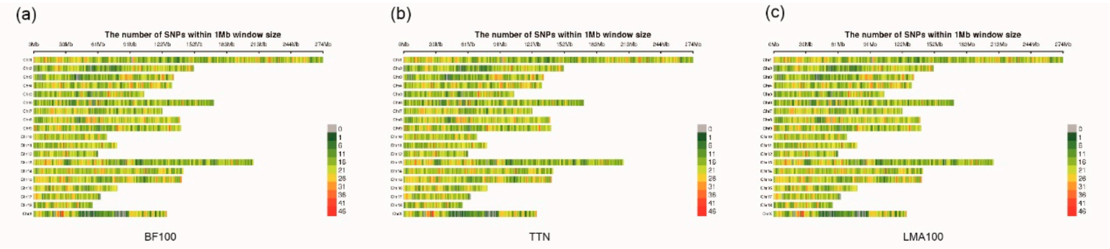

Supplementary Figure S1. SNP density plots for BF100, TTN, and LMA100 traits.
Heatmaps depict the number of SNPs within 1Mb window sizes across the genome for (a) backfat thickness at 100 kg (BF100), (b) total teat number (TTN), and (c) loin muscle area at 100 kg (LMA100). The color gradient indicates SNP density (red = high density, green = low density), illustrating the genomic distribution pattern of SNPs for each trait.

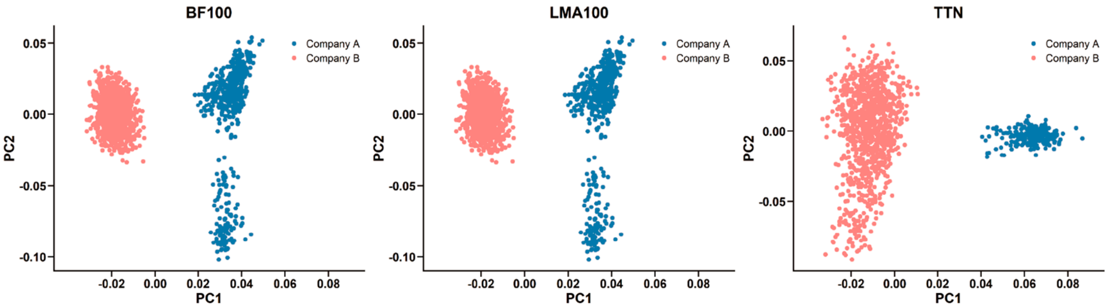

Supplementary Figure S2. Principal component analysis (PCA) of genomic variation in duroc pigs grouped by company origin.
Scatter plots depict the first two principal components (PC1 vs. PC2) for three economically important traits: backfat thickness at 100 kg (BF100), total teat number (TTN), and loin muscle area at 100 kg (LMA100).
